# Nanomechanical probing of the layer/substrate interface of an exfoliated InSe sheet on sapphire

**DOI:** 10.1038/srep26970

**Published:** 2016-06-03

**Authors:** Ryan Beardsley, Andrey V. Akimov, Jake D. G. Greener, Garry W. Mudd, Sathyan Sandeep, Zakhar R. Kudrynskyi, Zakhar D. Kovalyuk, Amalia Patanè, Anthony J. Kent

**Affiliations:** 1 School of Physics and Astronomy, The University of Nottingham, University Park, Nottingham NG7 2RD, UK; 2 School of Physics, Indian Institute of Science Education and Research Thiruvananthapuram (IISER-TVM), CET campus, Engineering College PO, Thiruvananthapuram, Kerala, India; 3 Frantsevich Institute for Problems of Materials Science, The National Academy of Sciences of Ukraine, Chernivtsi Branch, Chernivtsi 58001, Ukraine

## Abstract

Van der Waals (vdW) layered crystals and heterostructures have attracted substantial interest for potential applications in a wide range of emerging technologies. An important, but often overlooked, consideration in the development of implementable devices is phonon transport through the structure interfaces. Here we report on the interface properties of exfoliated InSe on a sapphire substrate. We use a picosecond acoustic technique to probe the phonon resonances in the InSe vdW layered crystal. Analysis of the nanomechanics indicates that the InSe is mechanically decoupled from the substrate and thus presents an elastically imperfect interface. A high degree of phonon isolation at the interface points toward applications in thermoelectric devices, or the inclusion of an acoustic transition layer in device design. These findings demonstrate basic properties of layered structures and so illustrate the usefulness of nanomechanical probing in nanolayer/nanolayer or nanolayer/substrate interface tuning in vdW heterostructures.

Metal monochalcogenide van der Waals layered crystals, such as InSe, GaSe and GaS, can be fabricated in the form of nanometer sheets with a thickness of several atomic monolayers. Recent reports of bendable photodetectors^1^, large-scale image sensors^2^, and field effect transistors (FETs) with large current on/off ratios (~10^8^) and high carrier mobilities (0.1 m^2^V^−1^s^−1^) at room temperature^3^ have demonstrated the potential of these layered compounds for future technologies. Also, because the layers are held together by weak van der Waals forces, both the structural integrity and the electronic properties of the individual atomic planes are maintained when they are combined with other two dimensional crystals (e.g. graphene) to create hybrid heterostructures^4^.

For the realization of these hybrid systems it is important to understand the properties of the interface between the semiconductor nanosheet and the substrate or a neighboring nanometer layer. Most of the studies performed on van der Waals heterostructures to date have been directed towards the measurement of the electron transport through the heterostructure or of the charge transfer at an interface^5^. However, the phonon transport through an interface plays an equally important role^6^,^7^ as it defines the cooling rate and so governs the thermal properties of a multi-layer structure. Specifically, the balance of electron and phonon transport between nanolayers quantifies the efficiency, *ZT*, in thermoelectric devices^8^,^9^,^10^. This is expected to be enhanced in metal chalcogenide thin layers due to the singularity in the density of states and the large number of conducting modes near the band edge^11^.

The microscopic origin of the phonon transport and thermal conductance through the interface of a nanodevice lies in the strength of the elastic bonds between atoms at the surfaces of two contacting materials. The interlayer phonon modes in 2D nanolayers have been studied using Raman scattering^12^. However, the bonds may not be perfect across the whole area of the nanolayer; in this case an effective way to study them is to monitor the nanomechanical vibrations of the sandwiched nanostructure^13^,^14^,^15^,^16^. Two dimensional (2D) nanolayers possess nanomechanical (i.e. phonon) resonances in the gigahertz (GHz) and terahertz (THz) frequency ranges. The frequency *f* and quality factor *Q* of these resonances depend on the elastic properties of the nanolayer/nanolayer or nanolayer/substrate interface, depending on the particular structure, and thus provide information about the elastic bonds at the interface.

In the present paper we use a picosecond acoustic technique to observe the coherent elastic vibrations of the fundamental thickness mode of InSe nanolayers on a sapphire substrate and to measure the values of *f* and *Q* for the nanomechanical acoustic phonon resonance. The comparison of experimental results with a simple theoretical analysis enables us to assess the mechanical adhesion of InSe on the substrate: we find that, acoustically, InSe layers with a thickness between 50 and 120 nm behave effectively as a free standing membrane with *Q* varying from 2 to 10 and *f* in the range of tens of gigahertz.

## Experiment and Results

**Figure 1:**
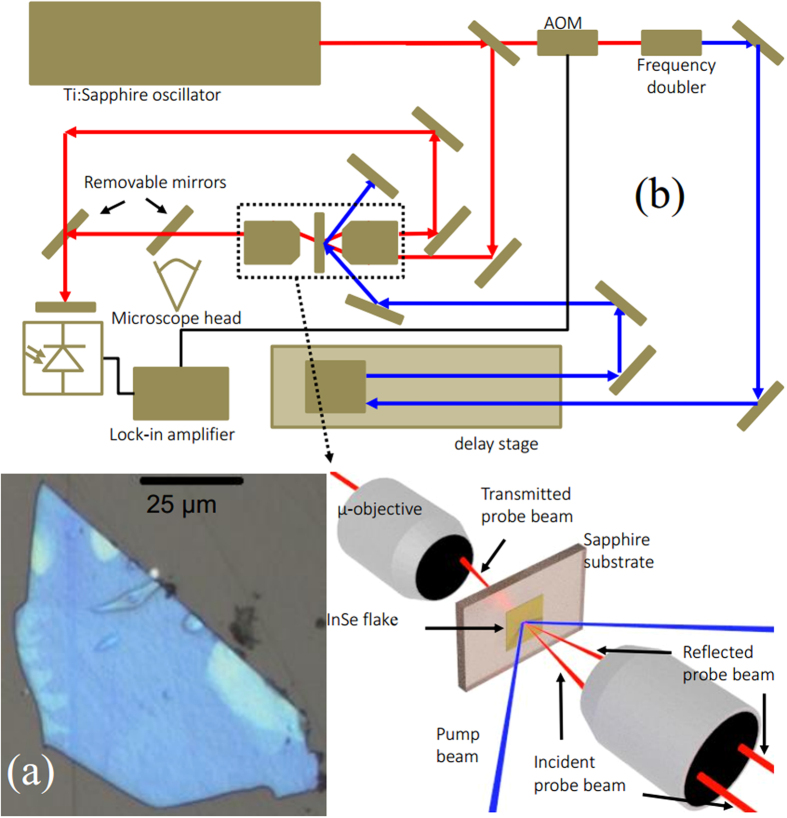
(**a**) The optical image of the studied InSe layer with a thickness of 114 nm exfoliated onto a sapphire substrate. (**b**) The experimental scheme of the pump-probe experiments.

InSe nanolayers were fabricated in the form of flakes by standard mechanical exfoliation from bulk Bridgman-grown crystals of γ-rhombohedral InSe. They were deposited onto a 200 μm thick sapphire substrate before soaking in acetone to remove the tape and residuals. The primitive unit cell of γ-rhombohedral InSe contains three stacks of InSe sheets. Each stack has a thickness of 8.320 Å and consists of four covalently bonded monoatomic sheets (Se-In-In-Se); along the *c*-axis, the primitive unit cell has a lattice constant of *c* = 24.961 Å and, within each *a-b* plane, atoms form hexagons with lattice parameter *a* = 4.002 Å. This crystal structure gives rise to a strong mechanical anisotropy. The van der Waals bonded sheets cleave cleanly during the exfoliation process leaving smooth flakes or flakes with multi-terraces. We use optical contrast to confirm the quality of the exfoliated InSe facet and tapping mode atomic force microscopy (AFM) to determine the thickness *d* of the fabricated flakes. An optical image of one of the flakes studied in the present work, with thickness *d* = 114 ± 2 nm, is shown in Fig. 1(a).

The main elements of the picosecond acoustic technique are shown in Fig. 1(b). Our time resolved optical instrument uses a mode locked Ti:Sapphire laser to generate 120 fs pulses with a central wavelength of 780 nm at a repetition rate of 82 MHz. The laser beam is partly reflected by a beam splitter to form the pump and probe paths. The probe beam is directed into a confocal microscope, consisting of two micro-objectives and a microscope head that can be removed from the beam path during the measurement. A removable mirror positioned before the detector allows the system to detect the probe beam that is either transmitted or reflected from the sample. The pump beam is modulated, frequency doubled and passed through a variable delay line to deliver pulses with a central wavelength of 390 nm to the sample and allow the stroboscopic measurement of the InSe flake. The pump and probe beams are focused to ~50 μm and ~5 μm, respectively, at the sample surface and the energy density of the pump beam does not exceed 0.5 μJcm^−2^. We use a band pass filter to prevent any photoluminescence (emitted from the sample at λ > 950 nm) from entering the detector. The photodiode signal is read by a lock-in amplifier locked to the modulation of the pump beam.

At the chosen wavelength there is strong absorption of the pump beam by the InSe flake, which is known to generate hot electron-hole pairs. These non-equilibrium carriers are coupled to the lattice via the electron-phonon interaction and thermoelastic effect, causing a local stress in the flake^17^. As a result of this stress, the InSe nanolayer starts to vibrate at its resonant frequency, which is defined by the boundary conditions and its thickness *d*. We monitor such vibrations by measuring the intensity variation of the transmitted or reflected probe beam, arising due to changes in the thickness of the nanolayer and the elasto-optical effect. We have measured more than 14 InSe nanolayers with thicknesses *d* between 20 and 200 nm. In the present study we focus on results obtained for a flake (Flake 1) with *d* = 114 nm, where the detection sensitivity for coherent vibrations is good and allows for a high signal to noise ratio.

**Figure 2:**
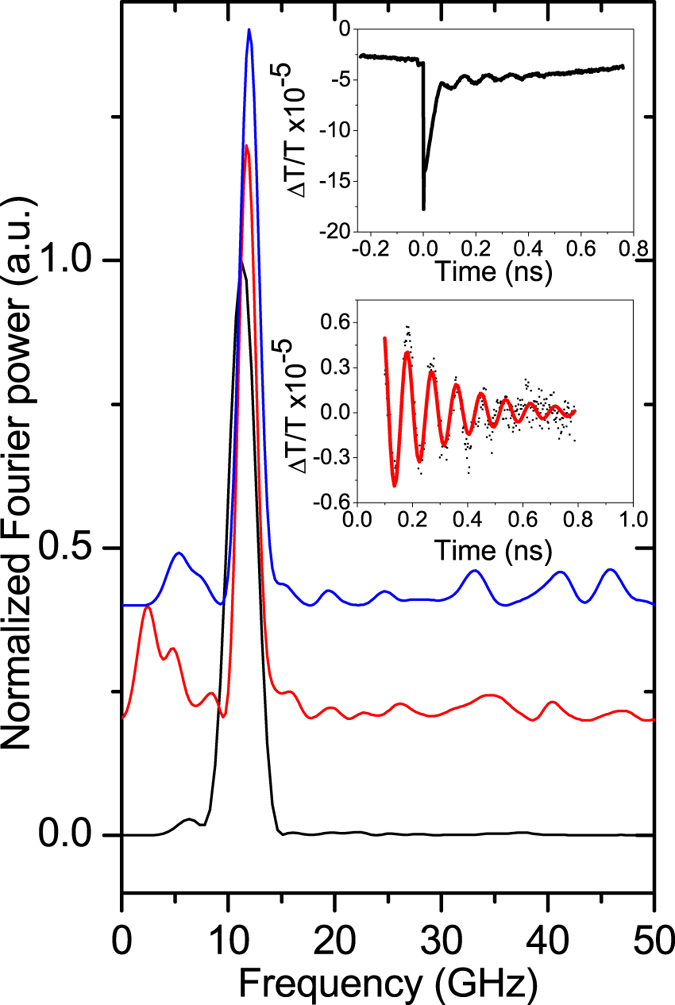
Power Fourier spectra of the pump-probe transmission signals measured in 3 locations on the InSe nanolayer with *d* = 114 nm. The upper and lower insets show an example of the temporal evolution of the measured signal before and after subtracting the slow decaying background, respectively. The solid line in the lower inset is the fit to the experimental data using Eq. (1).

**Figure 3:**
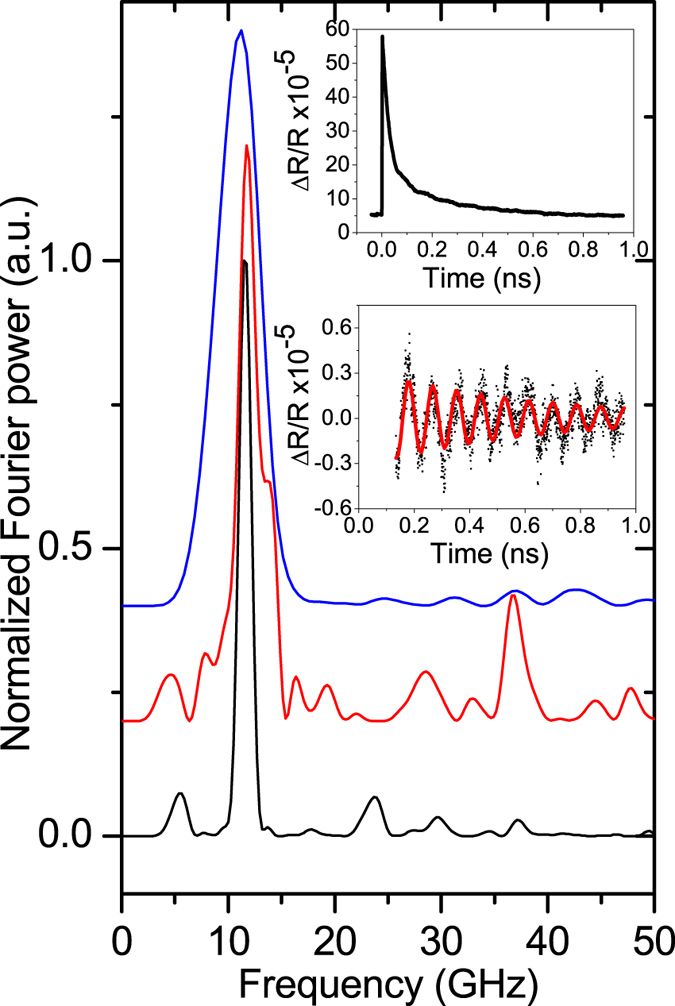
Power Fourier spectra of the pump-probe reflection signals measured in 3 locations on the InSe nanolayer with *d* = 114 nm. The upper and lower insets show an example of the temporal evolution of the measured signal before and after subtracting the slow decaying background, respectively. The solid line in the lower inset is the fit to the experimental data using Eq. (1).

Figures 2 and 3 show the results for transmission and reflection geometries, respectively. The upper insets show the temporal evolution of the pump-probe signals as a function of the delay, *t*, between pump and probe pulses. When pump and probe are concurrently incident on the sample, *i.e*. at *t* = 0, we observe a spike which is commonly attributed to the excitation of hot carriers by the pump pulse. The sign of this spike is negative in the transmission experiments and positive when the intensity of the reflected probe pulse is measured. The oscillatory behavior is clearly observed on the falling edges of the signals. The low insets of Figs 2 and 3 show these oscillations after the subtraction of the slow decaying background. Fast Fourier transforms (FFTs) of the subtracted signals are shown in the main panels Figs 2 and 3.

The solid lines in the lower insets in Figs 2 and 3 are the fits to the experimental data using the equation:


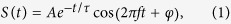


where *f, A, τ* and *φ* are the fitting parameters describing the frequency, amplitude, decay and initial phase of the oscillations. Fits to the data obtained in various locations on the InSe layer with Eq. (1) give *A*~10^−5^, *τ* = 0.2–0.9 ns, and *f* = 11.2–12.0 GHz. Measured values of *f* for flakes (Flakes 1–5) with different thickness *d* are shown in Table 1. It can be seen that the measured frequency *f* of the oscillations tends to increase with decreasing *d*. For flakes with *d* < 50 nm no oscillations were detected.

## Analysis and Discussion

Over the last decade similar oscillatory signals have been observed in picosecond acoustic pump-probe experiments with thin films both on substrates^18^,^19^,^20^,^21^ and free standing membranes^22^. These oscillations are attributed to the nanomechanical vibrations of the film in the direction perpendicular to the plane of the layer. Such vibrations are classified as the excitation of coherent longitudinal acoustic (LA) phonons and their phononic spectrum is quantized at the frequencies governed by the value of *d* and boundary conditions of the nanolayer at the interfaces. In the *free standing layers* (i.e. membranes) the surfaces are free and there the nodes take place for the deformation tensor elements while the displacements at both surfaces possess anti-nodes. The frequencies of these nanomechanical resonances are:


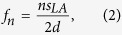


where *s*_*LA*_ is the longitudinal sound velocity in the direction perpendicular to the layer plane, *d* is the layer thickness and *n* is an integer. The mode with *n* = 1 corresponds to the modulation of the layer thickness and is known as fundamental thickness mode. The distribution of strain and displacement modes in such a layer are a harmonic function, equal to the half wavelength of the corresponding acoustic wave in the bulk. The quality factor *Q *= *πfτ* of the mechanical resonances, which are limited by the scattering of phonons and the inhomogeneity of the film thicknesses, may reach values up to several hundred^22^ in free standing membranes.

For *layers supported by a substrate*, with an ideal interface, the spectrum depends on the acoustic mismatch described by the differences in the acoustic impedances 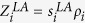 where the subscript *i* defines the material of the layer and *ρ*_*i*_ is its density. When the layer has lower impedance than the substrate, the frequencies of the nanomechanical resonances are:


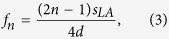


where *n* = 1, 2, …. When the layer impedance is higher than that of the substrate the equation for the resonance frequencies is the same as for the free standing membrane, shown in Eq. (2). The *Q*-factor of the resonances for supported nanolayers is governed by the reflection *r* of phonons at the interface:


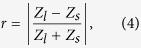


where the indexes *l* and *s* correspond to the layer and substrate, respectively. It is important to note that Eq. (4) is valid only for the case of perfect elastic contact between the layer and the substrate, i.e. an elastically perfect interface. If this condition is not met other sophisticated theoretical approaches^16^ should be used. For comparison of the experimental results with Eqs. (2)–(4), we use the following parameters for the InSe layer and sapphire (sap) substrate: 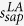 = 11 km/s, *ρ_sap_* = 4 g/cm^3^, 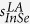 = 2.5 km/s^23^ and *ρ*_*InSe*_ = 5.5 g/cm^3^. For acoustic impedances we obtain: 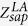 = 44 MPa∙s/m and 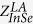 = 14 MPa∙s/m. Clearly, the acoustic impedance of the sapphire substrate is significantly higher than that of the InSe layer and so, in the case of an elastically perfect interface, the fundamental thickness mode (*n * = 1) should be calculated using Eq. (3). However, Flake 1 for instance, with *d* = 114 nm, gives a value of *f*_1_ = 5.5 GHz which is a factor of two smaller than the measured value of 11.6 GHz. The assumption that the experimental technique for our material system has greater sensitivity to higher harmonics also does not give an agreement. Eq. (3) for the next resonance, *n* = 2, gives *f*_2_ = 16.5 GHz, which is a factor of 1.4 larger than the measured value.

The only way to recover the measured value by calculation is to use Eq. (2), which corresponds to the case of a free standing InSe layer: the calculated value is *f*_1_ = 11.0 GHz which is in good agreement with the measured value. This agreement leads us to conclude that the interface between the InSe and the substrate should not be considered as a perfect elastic interface. Also, we estimate the reflectivity of acoustic phonons as *r* = exp(−π/*Q*). From the values of *Q* measured in various positions on the InSe nanolayer we obtain *r* = 0.64–0.91. Similar conclusion can be applied to other flakes, see Table 1. An excellent agreement between the measured and calculated values of *f* is obtained for Flakes 3 and 5. In contrast, small differences are noticed for flakes 1, 2 and 4. However, a more sophisticated theoretical analysis^16^,^24^ of the nanomechanical vibrations in the nanolayers shows that *f* depends on the elastic coupling parameter at the interface, thus resulting in values *f* that may differ from those corresponding to the extreme cases of weak and ideal elastic coupling, as described by Eq. (2) and Eq. (3), respectively.

The most likely reason for the imperfectness of the interface is surface roughness of the substrate rather than that of the InSe layer, which tends to form abrupt and atomically smooth interfaces with other layered compounds^25^. AFM measurements show the roughness of the substrate to be ~10 nm, which is higher than the thickness deviations in the InSe flake. To achieve an elastically perfect interface between the substrate and the exfoliated layer, the surfaces should be flat and clean down to the atomic level. The roughness of the substrate surface in our sample is far beyond this level. Thus it is unlikely that mechanical exfoliation of InSe on a rough substrate can provide an ideal elastic contact between the two materials. In this respect we would like to refer to experiments performed on MoS_2_ nanolayers exfoliated onto a Si/SiO_2_ substrate^26^. There the authors explore Eq.(3) above {Eq. (2) in ref. 26}, assuming an elastically perfect interface and obtain a sound velocity twice that obtained from the elastic constant, *c*_33_ = 52 GPa^27^, and density, *ρ*_*MoS2*_ = 5.06 gcm^−3^ of MoS_2_. If we assume that in the experiments reported in ref. 26 the interface is not elastically perfect and use Eq. (2), with 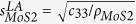, for *d* = 73 nm we find good agreement between the experimental (*f*_*1*_ = 24 GHz) and calculated (*f*_*1*_ = 22 GHz) values of *f*_1_. Thus, we think the nanomechanical resonance in MoS_2_ observed in ref. 26 results from an elastically imperfect interface between the nanolayer and the substrate, similar to the present case for InSe.

## Conclusion

In conclusion, the present work shows that an elastically imperfect interface forms between a sapphire substrate and an exfoliated van der Waals InSe crystal with thicknesses down to about 50 nm. With respect to the properties of interfaces between thinner flakes and/or other substrates, additional experiments are required, which is beyond the scope of this study, which demonstrates nanomechanical probing of an interface using coherent phonons.

Our findings suggest applications where the elastic properties of such an interface may be exploited: for example, layered structures with elastically imperfect interfaces may still possess efficient carrier transport and thus provide high *ZT* in thermoelectric nanodevices^8^,^9^,^10^. Alternatively, if the 2D nanolayer requires efficient thermal coupling to a substrate heat sink, the deposition of a soft nanometer transition layer (e.g. a polymer) on the substrate surface and annealing after the exfoliation may be included in the device fabrication procedure. The present work also indicates that nanomechanical probing of interfaces between 2D semiconductor layers and a substrate, or other nanolayers, could be successfully used for the study of elastic interface properties and hence the development of methods to control these interface properties.

## Additional Information

**How to cite this article**: Beardsley, R. *et al*. Nanomechanical probing of the layer/substrate interface of an exfoliated InSe sheet on sapphire. *Sci. Rep.* **6**, 26970; doi: 10.1038/srep26970 (2016).

## Figures and Tables

**Table 1:** Experimental results for nanomechanical probing in 5 InSe nanolayers.

Flake	Thickness, *d*, AFM (nm)	Frequency, *f*, (GHz)			
		Measured	Calculated		
			Eq. (2), *n* = 1	Eq. (3), *n* = 1	Eq. (3), *n* = 2
1	114 ± 2	11.6 ± 0.4	**11.0**	5.5	16.4
2	100 ± 4	13.3 ± 0.1	**12.5**	6.3	18.9
3	94 ± 5	13.4 ± 0.3	**13.3**	6.6	19.9
4	57 ± 4	26.2 ± 1.1	**21.9**	11.0	32.9
5	54 ± 3	23.1 ± 0.6	**23.1**	11.6	34.7

The measured values of *f* are obtained from the FFT spectra. The calculated values of *f* are obtained using Eq. (2) and Eq. (3). The calculated values marked by bold have the best agreement with the measured values. The results for Flake 1 are described in detail in the text.
