# Corrigendum: Nanomechanical probing of the layer/substrate interface of an exfoliated InSe sheet on sapphire

**DOI:** 10.1038/srep30528

**Published:** 2016-08-19

**Authors:** Ryan Beardsley, Andrey V. Akimov, Jake D. G. Greener, Garry W. Mudd, Sathyan Sandeep, Zakhar R. Kudrynskyi, Zakhar D. Kovalyuk, Amalia Patanè, Anthony J. Kent

Scientific Reports 6: Article number: 2697010.1038/srep26970; published online: 06 03 2016; updated: 08 19 2016.

The Acknowledgements section in this Article is incomplete.

“This work was supported by the Engineering and Physical Sciences Research Council (EPSRC), the EU FP7 Graphene Flagship Project 604391, the National Academy of Sciences of Ukraine, the Department of Science and Technology, India and the UK India Education and Research Initiative”.

should read:

“This work was supported by the Engineering and Physical Sciences Research Council [grant number EP/M012700/1]; the EU FP7 Graphene Flagship Project 604391; the National Academy of Sciences of Ukraine; the Department of Science and Technology, India; and the UK India Education and Research Initiative”.

